# Risk of chronic obstructive pulmonary disease in a large cohort of Ontario, Canada workers

**DOI:** 10.1038/s41598-024-59429-1

**Published:** 2024-04-16

**Authors:** Jeavana Sritharan, Victoria H. Arrandale, Tracy L. Kirkham, Mamadou Dakouo, Jill S. MacLeod, Paul A. Demers

**Affiliations:** 1 Occupational Cancer Research Centre, Ontario Health, 525 University Avenue, 3rd Floor, Toronto, ON M5G 1X3 Canada; 2 Dalla Lana School of Public Health, University of Toronto, Toronto, Canada

**Keywords:** COPD, Occupation, Sex differences, Surveillance, Respiratory conditions, Chronic obstructive pulmonary disease, Risk factors

## Abstract

Although several occupational exposures have been linked to the risk of COPD; limited data exists on sex-specific differences. This study aimed to identify at-risk occupations and sex differences for COPD risk. Cases were identified in a large surveillance system established through the linkage of former compensation claimants’ data (non-COPD claims) to physician visits, ambulatory care data, and hospital inpatient data (1983–2020). Cox proportional hazard models were used to estimate hazard ratios (HRs) and corresponding 95% confidence intervals (CI) for occupation groups (occupation at time of claim), stratified by sex. HRs were indirectly adjusted for cigarette smoking using another population dataset. A total of 29,445 male and 14,693 female incident cases of COPD were identified. Increased risks were observed in both sexes for construction (HR_male_ 1.15, 95% CI 1.12–1.19; HR_female_ 1.54, 95% CI 1.29–1.83) transport/equipment operating (HR_male_ 1.32, 95% CI 1.28–1.37; HR_female_ 1.53, 95% CI 1.40–1.68) farming (HR_male_ 1.23, 95% CI 1.15–1.32; HR_female_ 1.19, 95% CI 1.04–1.37) and janitors/cleaners (HR_male_ 1.31, 95% CI 1.24–1.37; HR_female_ 1.40, 95% CI 1.31–1.49). Increased risks were observed for females employed as chefs and cooks (HR 1.44, 95% CI 1.31–1.58), bartenders (HR 1.38, 95% CI 1.05–1.81), and those working in food/beverage preparation (HR 1.34, 95% CI 1.24–1.45) among other occupations. This study demonstrates elevated risk of COPD among both male and female workers potentially exposed to vapours, gases, dusts, and fumes, highlighting the need for occupational surveillance of COPD.

## Introduction

Chronic obstructive pulmonary disease (COPD) is a lung condition that is characterized by various chronic respiratory symptoms resulting from airway abnormalities such as bronchitis and/or emphysema^1^. It is one of the most common chronic respiratory diseases in Canada and continues to be a major cause of death and disability worldwide^2,3^. In 2021, the World Health Organization and International Labour Organization reported that COPD due to occupational exposure to particulate matter, gases, and fumes had the greatest number of work-related deaths and the third-highest number of work-related disability-adjusted life years (DALYs) worldwide^4^. The prevalence of COPD is higher in males when compared to females which may stem from underdiagnosis in females and historic differences in exposure potentially leading to an underestimation of the current burden of disease in females^5^.

Tobacco smoking is the largest established cause of COPD^6^. However, it is estimated that 25–45% of people with COPD never smoked, which emphasizes the importance of examining other potential risk factors^3,7^. Other possible causes include history of childhood respiratory conditions (e.g., asthma), air pollution, occupational exposures (including second hand smoke)^6,8^.

Occupational exposures are an under-appreciated risk factor for COPD^1^. Based on evidence showing that occupational exposure to vapours, gases, dusts, and fumes (VGDF) increases the risk of COPD^9–11^, the American Thoracic Society and European Respiratory Society estimated that workplace exposures contribute to 14% of all COPD cases^8^. Increased risk of COPD has been observed among specific groups such as construction workers^11–13^, and farm and wood industry workers^14^. COPD has been also linked to a wide range of specific substances common in certain workplaces, including cotton dust, farm dust, grain dust, wood dust, welding fumes, and crystalline silica^9^. Other exposures linked to COPD may include diesel engine exhaust^11^, pesticides^15,16^, organic dust^14^, and second-hand smoke^11^. Workers can be exposed to multiple overlapping occupational exposures which poses a challenge in separating out individual exposures related to risk of COPD.

This study utilizes a unique surveillance system that contains physician billing, hospital discharge, and emergency room records to examine the risk of COPD in a wide range of occupations. Additionally, this study addresses a notable gap in the current body of literature by exploring potential sex differences in COPD risk. The overarching objective of this study was to examine and compare the risk of COPD by occupation in males and females in Ontario.

## Methods

This study uses the Occupational Disease Surveillance System (ODSS) which was established to monitor work-related diseases among approximately 2.3 million Ontario workers. The ODSS was created by linking workers with accepted lost time compensation claims (non-COPD claims) between 1983 to 2019 identified through the Ontario Workplace Safety and Insurance Board (WSIB). Lost-time claims pertain to work-related injuries or diseases leading to time away from work, loss of wages or earning capacity, or permanent impairment or disability. The WSIB provides coverage to approximately 70–75% of Ontario workers^17^, and accepted claims records demonstrate an overrepresentation of workers in hazardous sectors such as construction, transportation, and manufacturing where workers are more likely to be injured. About 95% of claims involve trauma and musculoskeletal injuries. The WSIB claims data continues to be a crucial source of work-related information for linked cohorts such as the ODSS. The workers’ occupation(s) were coded by WSIB at the time of each claim using the 1971 Canadian Classification Dictionary of Occupations (CCDO). The CCDO is organized in a structure that allows occupations to be classified at three levels: division, major, and minor. Division level occupations are the broadest categories, followed by the major level occupations which further subdivide the jobs, followed by the minor level occupations which allows for a more detailed and granular classification of jobs. Workers with accepted lost-time claims were eligible for linkage to the Registered Persons Database (RPDB) (1990–2022) which contains information on demographic factors, death/emigration out of province, and residence. The RPDB also contains a unique identifier, known as the health insurance number (HIN), for all Ontarians registered for insured health services. A total of 1,973,312 workers linked to the RPDB had a corresponding HIN. Those without a HIN were excluded from the analytical cohort.

By use of HIN, workers were linked to physician billing (OHIP), ambulatory care data (National Ambulatory Care Reporting System, NACRS), and hospital inpatient data (Discharge Abstract Database, DAD) to identify incident cases of COPD. Cases were defined as those who had three or more OHIP records for COPD (International Classification of Diseases, Ninth Revision, 491–492 or 496) within two years, or three or more NACRS records (ICD-10 J41-J44) within two years, or one DAD record (ICD-10 J41-J44) at any time. To capture the exposure window of the working population and COPD diagnosis, the cohort was restricted to workers aged 35–65 years (age at time of accepted claim), with a total of 1,565,259 workers included in this analysis. As a result, 44,138 cases of COPD were identified in this analysis. Males and females with COPD were analyzed separately.

Workers were followed from the date of their first WSIB accepted claim (entry into cohort) to the date of first diagnosis, age 65 years, emigration out of Ontario, death, or end of study (December 31, 2020). Cox proportional hazard models were used to estimate hazard ratios (HR) with 95% confidence intervals (CI). All models were adjusted for age at the start of follow-up (continuous) and birth year (continuous). These covariates were selected prior to all analyses with the ODSS to reduce confounding due to age, birth cohort effects, and sex differences among employment and disease risk. For all models, risk of COPD among workers in each occupation group was compared to all other workers in the ODSS. For example, workers employed in construction trades were compared to all other workers in all other occupations in the ODSS.

The analysis was repeated with the removal of workers who had an asthma diagnosis prior to or at the same time as a COPD diagnosis (n = 27,715). Further, cigarette smoking prevalence estimates were assessed for division level industry groups obtained from eight pooled cycles of the Canadian Community Health Survey (CCHS) (2007–2014) based on Ontario respondents only. Stratums were created by grouping CCHS participants by their North American Industry Classification System (NAICS) code, sex, and birth year (as 5-year age groups). The prevalence of current smokers was calculated for each stratum. As the ODSS uses the Standard Industrial Classification (SIC), different from the coding system used in the CCHS data; a crosswalk was applied between the two datasets. This allowed for application of the indirect adjustment to broad level occupation groups in the ODSS. Two models are shown with adjustment for age at start of follow up and birth year, followed by adjustment for age at start of follow up, birth year, and cigarette smoking.

All analyses were conducted in SAS 9.4 (SAS Institute, Cary, NC, USA). Ethics approval was granted by the University of Toronto Health Sciences Research Ethics Board (REB). All methods adhere to relevant guidelines and regulations under the provision of Ontario Health and the approved REB. Informed consent is not required for secondary data use which is also under the provision of Ontario Health (where the research took place) and the approved REB (reference 39013).

## Results

### Cohort characteristics

Among approximately 1.5 million workers in the analytical cohort, 44,138 incident cases of COPD were identified, with 29,445 cases among males and 14,693 cases among females. A comparison of COPD cases and the overall analytical cohort by sex is shown in Table 1.

**Table 1:** Characteristics of COPD cases and the overall ODSS cohort (1983–2020).

	COPD cases		ODSS overall	
	Males	Females	Males	Females
Total	29,445	14,693	1,040,009	525,250
Year of birth Median (IQR)	1955 (1950–1961)	1955 (1950–1960)	1962 (1954–1970)	1961 (1953–1969)
Years of follow-up Median (IQR)	5 (2–8)	4 (2–8)	12 (6–14)	10 (5–14)
Age at start of follow-up Median (IQR)	51 (46–56)	51 (46–56)	45 (37–52)	44 (39–55)
Age at end of follow-up Median (IQR)	57 (51–61)	57 (52–61)	57 (50–65)	59 (51–65)

*IQR* Interquartile Range, *COPD* chronic obstructive pulmonary disease, *ODSS* Occupational Disease Surveillance System.

### COPD risk by occupation group

Table 2 presents sex-stratified hazard ratios for all division level occupation groups and select major level occupation groups. Major level occupation groups were included in Table 2 if either sex demonstrated an elevated risk for COPD at the division level. Minor level occupation groups are shown in Supplementary Table 1. Sex-stratified estimates for division-level occupation groups with two different models, the first with adjustment for age at start of follow up and birth year and the second model with adjustment for age at start of follow up, birth year, and cigarette smoking are shown in Table 3. We also tested for interaction between sex and occupation in our modelling and p-values are shown in Table 2. Additionally, a sensitivity analysis was performed to exclude workers who had a prior asthma diagnosis, and findings are shown in Supplementary Table 2. Results show that only nine risk estimates slightly attenuated but did not impact overall results. For all analyses, workers in each occupation group were compared to all other workers in the ODSS.

**Table 2:** Risk of COPD by division and select major level occupation group in males and females in the ODSS (1983–2020).

Occupation group (division and major levels)	Males		Females		P value**
	Cases	HR (95% CI)*	Cases	HR (95% CI)*	
Managers and administration	245	*0.72 (0.64–0.82)*	258	*0.68 (0.60–0.76)*	0.4064
Natural sciences, engineering, mathematics	364	*0.74 (0.67–0.82)*	98	0.85 (0.69–1.03)	0.3269
Social sciences and related	115	*0.80 (0.67–0.96)*	432	0.94 (0.86–1.04)	0.0991
Teaching and related	103	*0.41 (0.34–0.50)*	330	*0.35 (0.32–0.39)*	0.1885
Medicine and health	276	*0.76 (0.68–0.86)*	2028	*0.77 (0.74–0.81)*	0.9634
Artistic, literacy, and recreational	137	0.88 (0.75–1.05)	90	0.91 (0.74–1.12)	0.7280
Clerical and related	2125	1.02 (0.97–1.06)	2162	1.04 (0.99–1.09)	0.3801
Services	3413	0.98 (0.95–1.02)	3906	**1.32 (1.27–1.37)**	< 0.0001
Protective services	673	*0.60 (0.56–0.65)*	185	1.03 (0.89–1.19)	< 0.0001
Food and beverage preparation and related services	634	0.95 (0.88–1.03)	1655	**1.40 (1.33–1.47)**	< 0.0001
Sales	1134	0.97 (0.91–1.03)	1422	**1.07 (1.01–1.13)**	0.0044
Farming, horticulture, and animal husbandry	799	**1.23 (1.15–1.32)**	197	**1.19 (1.04–1.37)**	0.7440
Farmers	30	1.13 (0.79–1.62)	8	1.11 (0.56–2.22)	0.9818
Other farming, horticultural and animal husbandry	777	**1.24 (1.15–1.33)**	192	**1.20 (1.04–1.39)**	0.6824
Fishing, hunting, and trapping	16	1.56 (0.95–2.54)	< 6	–	0.3753
Forestry and logging	247	**1.29 (1.14–1.47)**	< 6	–	0.1965
Mining and quarrying	330	**1.23 (1.10–1.37)**	< 6	–	0.3826
Processing (mineral, metal, chemical)	1735	**1.24 (1.18–1.30)**	501	**1.39 (1.27–1.52)**	0.0091
Mineral ore treating	34	**1.54 (1.10–2.15)**	< 6	–	0.7214
Metal processing and related	786	**1.30 (1.21–1.40)**	65	**1.40 (1.10–1.79)**	0.5274
Clay glass and stone processing forming and related	248	**1.25 (1.10–1.42)**	44	**1.77 (1.32–2.38)**	0.0064
Chemicals petroleum rubber plastic and related materials processing	712	**1.13 (1.05–1.22)**	397	**1.35 (1.22–1.49)**	0.0024
Processing (food, wood, textile)	1556	**1.15 (1.09–1.21)**	774	**1.17 (1.09–1.26)**	0.5615
Food and beverage and related processing	931	1.03 (0.97–1.10)	587	**1.17 (1.07–1.27)**	0.0254
Wood processing except paper pulp	173	**1.39 (1.20–1.62)**	19	1.16 (0.74–1.81)	0.4789
Pulp and papermaking and related	154	**1.31 (1.12–1.54)**	17	0.98 (0.61–1.57)	0.2301
Textile processing	162	**1.33 (1.14–1.55)**	109	1.14 (0.95–1.38)	0.2663
Other processing	179	**1.53 (1.32–1.78)**	48	**1.37 (1.03–1.82)**	0.7799
Machining and related	4191	**1.09 (1.05–1.12)**	708	**1.49 (1.39–1.61)**	< 0.0001
Metal machining	797	*0.84 (0.78–0.90)*	96	**1.59 (1.30–1.94)**	< 0.0001
Metal shaping and forming except machining	3154	**1.16 (1.12–1.20)**	559	**1.50 (1.38–1.64)**	< 0.0001
Wood machining	179	1.09 (0.94–1.26)	28	1.09 (0.76–1.59)	0.9443
Clay, glass, stone and related materials machining	47	1.27 (0.96–1.69)	< 6	–	0.9147
Other machining and related	325	**1.17 (1.05–1.30)**	56	**1.68 (1.29–2.19)**	0.0060
Product fabricating, assembling, and repairing	6058	0.99 (0.96–1.02)	1811	**1.28 (1.21–1.34)**	< 0.0001
Fabricating and Assembling Metal Products, n.e.c	1852	**1.08 (1.03–1.13)**	637	**1.44 (1.33–1.56)**	< 0.0001
Fabricating assembling installing and repairing electrical and electronic and related equipment	513	0.84 (0.77–0.92)	334	**1.23 (1.11–1.38)**	< 0.0001
Fabricating assembling and repairing, wood	511	**1.14 (1.05–1.25)**	80	**1.33 (1.07–1.66)**	0.0506
Fabricating assembling and repairing, textile fur and leather	197	1.01 (0.88–1.16)	332	*0.88 (0.79–0.98)*	0.0754
Fabricating assembling and repairing, rubber, plastic, and related	342	**1.28 (1.15–1.42)**	136	**1.49 (1.26–1.76)**	0.0966
Mechanics and repairers except electrical	2354	*0.90 (0.86–0.94)*	82	**1.63 (1.31–2.03)**	< 0.0001
Other product fabricating assembling and repairing	934	**1.16 (1.09–1.24)**	387	**1.42 (1.28–1.57)**	0.0003
Construction trades	4820	**1.15 (1.12–1.19)**	126	**1.54 (1.29–1.83)**	0.0009
Excavating grading paving and related	555	**1.35 (1.25–1.47)**	16	**1.67 (1.02–2.73)**	0.3360
Electrical power lighting and wire communications equipment erecting installing and repairing	557	*0.65 (0.60–0.71)*	23	0.86 (0.57–1.29)	0.2169
Other construction trades	3829	**1.27 (1.23–1.31)**	88	**1.89 (1.54–2.34)**	< 0.0001
Transport and equipment operating	4534	**1.32 (1.28–1.37)**	470	**1.53 (1.40–1.68)**	0.0039
Air transport operating	75	*0.54 (0.43–0.67)*	16	*0.61 (0.37–0.99)*	0.7089
Railway transport operating	116	1.17 (0.97–1.40)	6	1.31 (0.59–2.91)	0.7224
Water transport operating	68	**1.49 (1.18–1.90)**	< 6	–	0.6839
Motor transport operating	4133	**1.38 (1.34–1.43)**	335	**1.70 (1.53–1.90)**	< 0.0001
Other transport and related equipment operating	322	1.06 (0.95–1.19)	153	**1.49 (1.27–1.75)**	0.0005
Materials handling and related	3220	**1.33 (1.28–1.38)**	846	**1.36 (1.26–1.45)**	0.3483
Other crafts and equipment operating	572	**1.09 (1.01–1.19)**	205	**1.48 (1.29–1.70)**	< 0.0001
Printing and related	350	1.09 (0.98–1.21)	190	**1.59 (1.37–1.83)**	< 0.0001
Stationary engine and utilities equipment operating and related	201	1.10 (0.96–1.26)	13	1.46 (0.85–2.52)	0.3034

Division level occupations are shown with a underline, major level occupations are indented.

Statistically significant (α = 0.05) increased risks are bolded, and statistically significant decreased risks are italicized.

*COPD* chronic obstructive pulmonary disease, *ODSS* occupational disease surveillance system, *n.e.c.* not elsewhere classified.

*Adjusted for birth year and age at start of follow up.

**The p-value demonstrates the test for interaction between sex and occupation.

**Table 3:** Risk of COPD by occupation group in males and females in the ODSS (1983–2020) with indirect adjustment for cigarette smoking.

Occupation group (division level)	Males			Females		
	Cases	Model 1 HR (95% CI)*	Model 2 HR (95% CI)^†^	Cases	Model 1 HR (95% CI)*	Model 2 HR (95% CI)^†^
Managers and administration	245	*0.72 (0.64–0.82)*	*0.75 (0.66–0.85)*	258	*0.68 (0.60–0.76)*	*0.69 (0.61–0.78)*
Natural sciences, engineering, mathematics	364	*0.74 (0.67–0.82)*	*0.76 (0.68–0.84)*	98	0.85 (0.69–1.03)	0.84 (0.69–1.03)
Social sciences and related	115	*0.80 (0.67–0.96)*	0.85 (0.71–1.02)	432	0.94 (0.86–1.04)	0.99 (0.90–1.09)
Teaching and related	103	*0.41 (0.34–0.50)*	*0.43 (0.35–0.52)*	330	*0.35 (0.32–0.39)*	*0.37 (0.33–0.41)*
Medicine and health	276	*0.76 (0.68–0.86)*	*0.80 (0.71–0.90)*	2028	*0.77 (0.74–0.81)*	*0.81 (0.77–0.85)*
Artistic, literacy, recreational	137	0.88 (0.75–1.05)	0.93 (0.78–1.10)	90	0.91 (0.74–1.12)	0.93 (0.76–1.15)
Clerical and related	2125	1.02 (0.97–1.06)	1.02 (0.97–1.06)	2162	1.04 (0.99–1.09)	1.02 (0.98–1.07)
Sales	1134	0.97 (0.91–1.03)	0.97 (0.92–1.03)	1422	**1.07 (1.01–1.13)**	1.03 (0.97–1.09)
Services	3413	0.98 (0.95–1.02)	**1.05 (1.01–1.09)**	3906	**1.32 (1.27–1.37)**	**1.42 (1.36–1.47)**
Farming, horticultural, animal husbandry	799	**1.23 (1.15–1.32)**	**1.28 (1.19–1.38)**	197	**1.19 (1.04–1.37)**	**1.23 (1.07–1.41)**
Fishing, hunting, trapping	16	1.56 (0.95–2.54)	1.58 (0.97–2.58)	< 6	–	–
Forestry and logging	247	**1.29 (1.14–1.47)**	**1.31 (1.16–1.49)**	< 6	–	–
Mining and quarrying, including oil and gas	330	**1.23 (1.10–1.37)**	**1.13 (1.01–1.26)**	< 6	–	–
Processing (mineral, metal, chemical)	1735	**1.24 (1.18–1.30)**	**1.22 (1.16–1.28)**	501	**1.39 (1.27–1.52)**	**1.30 (1.19–1.42)**
Processing (food, wood, textile)	1556	**1.15 (1.09–1.21)**	**1.14 (1.09–1.20)**	774	**1.17 (1.09–1.26)**	**1.11 (1.03–1.19)**
Machining and related	4191	**1.09 (1.05–1.12)**	**1.07 (1.04–1.11)**	708	**1.49 (1.39–1.61)**	**1.40 (1.30–1.51)**
Product fabricating, assembling, repairing	6058	0.99 (0.96–1.02)	0.98 (0.95–1.01)	1811	**1.28 (1.21–1.34)**	**1.20 (1.14–1.26)**
Construction trades	4820	**1.15 (1.12–1.19)**	**1.11 (1.08–1.15)**	126	**1.54 (1.29–1.83)**	**1.48 (1.24–1.76)**
Transport equipment and operating	4534	**1.32 (1.28–1.37)**	**1.32 (1.28–1.36)**	470	**1.53 (1.40–1.68)**	**1.47 (1.34–1.61)**
Materials handling and related	3220	**1.33 (1.28–1.38)**	**1.32 (1.28–1.37)**	846	**1.36 (1.26–1.45)**	**1.29 (1.20–1.38)**
Other crafts and equipment operating	572	**1.09 (1.01–1.19)**	**1.10 (1.01–1.20)**	205	**1.48 (1.29–1.70)**	**1.41 (1.23–1.62)**

*Model 1: adjusted for birth year and age at start of follow up.

^**†**^Model 2: adjusted for birth year, age at start of follow up, and cigarette smoking (based on industry level prevalence estimates).

Overall, this study identified patterns of increased risk of COPD in division-level (broad) occupations such as construction, transportation, farming, forestry, mining, and processing (involves manual tasks such as material handling, packaging, and other elemental activities) among both male and female workers. Other groups with elevated risk of COPD among both sexes included machining; product fabricating, assembling, and repairing; and materials handling (Table 2). Indirect adjustment for cigarette smoking showed no significant change in risk estimates across division level occupations (Table 3). At the major level, various occupations demonstrate increased risk of COPD for males and females as shown in Table 2. A number of occupations at the division-level also demonstrated decreased risks for males and females such as managerial and administration, teaching and related, and medicine and health occupations (Table 2). These findings remained consistent with indirect adjustment for cigarette smoking (Table 3).

There were also many minor-level groups across occupation with similar risk estimates for males and females shown in Supplementary Table 1. Some examples include construction trades occupations (e.g., excavating, grading and related; painters and paperhangers), transportation and equipment operating (e.g., truck driving; motor transport operating), farming (e.g., farm workers), machining (e.g., metal-working machine operating, welding and flame cutting), product fabricating, assembling and repairing (e.g., motor vehicle fabricating and assembling), processing related to mineral/metal/chemical (e.g., moulding and coremaking; mixing and blending), and processing related to food/water/textile (e.g., baking and confectionary making).

Multiple occupations showed an elevated risk of COPD only for males, at all three levels (division, major, and minor). This included occupations in construction trades (e.g., insulators; structural metal erectors; glaziers), transportation (e.g., water transport operating, boiler room crew), farming (e.g., nursery and related work), forestry and logging (e.g., timber cutting), wood processing (sawmill sawyers), mining and quarrying (e.g., cutting, handling, and loading), and processing (e.g., metal smelting, textile finishing and calendaring). It is important to note that many of these groups are predominately male with less than six cases among female workers and could not be reported.

There were also elevated risks identified for only female workers. Elevated risks were observed in construction trades (e.g., plastering and pipefitting/plumbing), transportation and equipment operating (e.g., motor transport operating such as bus and taxi driving), processing (e.g., clay, glass and stone processing and forming; textile winding, reeling and knitting) (Supplementary Table 1).

Although males and females had similar risks in machining and product fabricating, assembling, and repairing at all three occupational levels, there were some differences at the minor level. Males working in machining as boilermakers, platers, or in structural metal work had increased risks for COPD, whereas the risk was elevated among females employed as machine tool operators. For product fabricating, assembling, and repairing, males employed in bonding and cementing of rubber and plastic products and females employed in motor vehicle mechanics and repair and painting and decorating had elevated risks.

Both male and females employed as janitor, charworkers (light duty cleaning such as in offices, hotels, private homes, etc.), and cleaners had increased risks of COPD. However, unlike males, females employed across many service occupations showed elevated risks, such as guards and watchmen, chefs and cooks, bartenders, waitresses/hostesses/stewardesses, food and beverage preparation services, supervisors in lodging and accommodation, managers in hotels/motels and other accommodations, personal services, pressing occupations, supervisors in other services work, and other services not elsewhere classified (Supplementary Table 1).

## Discussion

Increased risk of COPD was observed among workers in construction, transportation, farming, forestry, mining, and processing. Patterns for these jobs, where common exposures include organic and inorganic dust and diesel exhaust, were similarly increased for males and females. Notable sex differences in risk of COPD were also identified in this study, particularly among service occupations. This study attempted to indirectly adjust for smoking by applying prevalence estimates that were generated using provincial population survey data. Findings showed that there were no significant differences in risk when adjusting for cigarette smoking. Although this is not based on the true smoking status of workers in the ODSS cohort, this study sought out a method to include population smoking data to address cigarette smoking.

This study found that the risk among construction workers (predominately male) was driven by those employed in excavating, grading and related; concrete finishing and related; painting, paperhanging and related; insulation; roofing, waterproofing and related; structural metal erecting; glazier work; labour and elemental work; and other construction trades (e.g., brick and stone masons, carpenters, plasters). Similar to this study, a recent US study reported a 34% increased risk of COPD among construction workers compared to non-construction workers^12^. They reported the highest risks among roofers (122% increased risk) and cement masons/bricklayers (136% increased risk)^12^. Another study in the UK observed higher risks of COPD among roofers, floorers and labourers, but not for masons or bricklayers^18^. It is likely that these workers are exposed to respirable dust given the nature of their work.

The excess risk observed among insulators in this study may be due to exposures during the application, removal and repair of thermal shields in buildings and pipes, including insulating materials, gypsum, and asbestos. A recent study of Canadian insulators reported an association (44% increased risk) between asbestos exposure and COPD^19^. A Swedish study reported finding a positive exposure–response relationship among males exposed to particles from gypsum and insulation material (56% increased risk)^20^. We observed an increased risk among female plumbers and pipefitters who may also be exposed to asbestos containing materials, inhalable dusts, and welding fumes^21^. Other construction work (e.g., glazing, metal erecting) where increased risks were noted, may involve exposure to respirable dust leading to the development of COPD^21,22^.

Workplace secondhand smoke (SHS) exposure has also been shown to be higher in non-smoking construction workers^23^. Studies have shown that when adjusting for smoking history or restricting to never smokers, there was little or no impact on risk estimates^14,18,24^.

This study identified increased risks of COPD among male water and motor transport workers and female motor transport workers (e.g., bus, taxi, truck drivers). The evidence on risk of COPD among transport workers is limited. Previous studies have speculated that risk of COPD in transportation, motor vehicle operators, and vehicle mechanics is related to air pollution, diesel engine exhaust exposure, among other exposures (second-hand smoke, dust, etc.,)^25–27^. The Swedish study identified a significant positive exposure–response trend for COPD among males exposed to diesel engine exhaust (18% increased risk)^20^. Another US study showed an increased risk for motor-vehicle operators and material moving workers (60% increased risk) and findings remained consistent when restricted to never smokers (50% increased risk)^27^. A large cohort study examined the association of exposure of fine particulate matter (PM_2.5_) and the prevalence and incidence of various chronic diseases among Canadian women. They reported elevated prevalence (12% increased risk) and incidence (17% increased risk) of COPD, with adjustment for smoking history^28^. As expected, the prevalence of COPD was much higher among smokers in their study^28^. Furthermore, SHS exposure in transportation workers is also possible as these workers are often outdoors or within idling vehicles in areas where smoking may not be prohibited.

This study identified an increased risk among farming workers driven by two groups, farm workers and nursery and related workers. Increased risks in farming may be due to exposure to various agents such as PM_2.5_, inhalable PM, and endotoxins found in dusts^29,30^. A study of animal farmers in the European Farmers’ Study found that dust and endotoxins demonstrated a dose–response relationship with COPD among never-smokers^31^.

An increased risk of COPD was observed for males employed in forestry and wood processing, which may be due to wood dust exposure, a known cause of adverse respiratory symptoms^32,33^. Conversely, a study on Danish farming and wood industry workers found an inverse association with COPD with cumulative organic dust exposure^14^. Other respiratory irritants such as ozone and chlorine dioxide/sulphur dioxide gassings are likely exposures among specific groups, such as pulp mill workers^34^.

Male mining workers demonstrated an increased risk of COPD driven by few groups, such as other rock and soil-drilling and various mining and quarrying work. Reduced lung function among these workers may be due to exposure to silica/mineral dust^22,35–37^ and diesel engine emissions^36^. A recent US study observed a higher COPD prevalence among mining industry workers, stronger among never smokers^27^. Another US study, the Diesel Exhaust in Miners Study, examined mortality from COPD and identified a stronger association among surface miners than among underground miners^36^.

Various processing occupations had increased risks for COPD in males and females. Processing occupations encompass a range of jobs and our study identified elevated risks among mineral oil treating; metal processing; clay, glass, stone processing; and chemical, petroleum, rubber, plastic and related materials processing. Exposure to mineral dust, adjusted for smoking, has been shown to be associated to risk of COPD^22^ which may be an exposure in processing occupations. Workers in textile processing may be exposed to various organic and synthetic dusts. Specifically, workers exposed to cotton dust long term may be at risk for respiratory symptoms^38^. There are large variations in exposure to inhalable dusts and airborne endotoxins, which may be depend on the dustiness of the work environment^39^, and the mechanisms involved remain unclear^38^.

Baking and confectionery making was elevated similarly for men and women in our study (19% and 24% respectively), although only slightly elevated for flour and grain milling in males (unreportable in females). Flour dust is a common exposure in bakeries and confectioneries, among other food related occupations, and may contain contaminants such as endotoxins, fungi, chemical additives, and insects and mites^40^. Although no peer-reviewed studies reporting on the risk of flour dust and COPD have been identified, flour dust exposure has been associated with an increase in respiratory symptoms^40,41^.

Risk of COPD was elevated across various machining occupations among males and females, including metalworking-machine operating, welding, metal shaping and forming. Specifically among females, increased risks were observed in occupations such as machine tool operating and motor vehicle mechanics and repairing. These jobs involve a wide range of potential exposures, including welding fumes, among other metal fumes/dusts and solvents^26^. Notably, welding fumes have been associated with COPD, showing a 57% increased risk among workers in the Swedish study^20^. Another study that examined risk of COPD among Korean shipyard workers identified an elevated risk among workers with middle and high welding fume exposure, when compared to the low fume exposure group^42^.

Within service occupations, there were interesting findings among female workers in this study, although there is limited evidence in the existing literature. A recent US study reported increases among both male and females employed as cooks and food preparation workers, food and beverage servers, and personal care service workers^27^. However, when they restricted to never smokers, findings attenuated^27^. Our study also identified increased risks for both sexes in janitor and cleaning occupations, similarly, a European study identified elevated risks across various cleaning occupations^18^. Our study also identified an elevated risk among laundering and dry-cleaning male workers, with only a slight increase among female workers.

We observed reduced risks for males and females across medicine and health occupations, however at the minor level, females employed as nursing aides and orderlies had a 9% increased risk of COPD. There is little evidence to support an increased risk of COPD among nurses. A previous study on a cohort of US female nurses reported that weekly use of any disinfectants was positively associated with COPD incidence. The corresponding population-attributable fraction was 12%^43^.

Understanding sex differences, specifically the risk among females, can be quite challenging. Past studies have faced difficulties in examining risk of COPD among female workers due to limited sample sizes, insufficient occupational history, and other constraints in understanding potential exposure differences by sex. Differences observed in our study may stem from true differences in exposure by sex, but may also be influenced by other factors such as gender biases, personal protective equipment effectiveness, and lifestyle or other non-occupational factors (e.g., individual cigarette smoking history)^44–46^.

There are several limitations in this study. Occupation information is only captured at a single point in time without complete work history or employment duration, which may contribute to non-differential misclassification. The study also lacks information on demographic characteristics (e.g. socioeconomic status, race, individual smoking status), other than sex and age, although occupation is often used as an indicator of socioeconomic status. We indirectly adjusted for cigarette smoking which can lead to possible residual confounding through the use of broad industry groups and the need for a crosswalk between job classifications. SHS exposure may vary across occupational groups and a recent study identified SHS exposure in Canadian workers was highest among trades, transport, equipment operators and primary industry workers, although rates were lowest in Ontario compared to other Canadian provinces^47^. Differences in SHS exposure may be related to challenges in workplace smoking policy adherence, unclear work guidelines for where smoking is permitted (e.g. in buildings under construction), lack of legislation for outdoor work environments, or higher smoking rates in trades work^47^, although we could not account for SHS in this study.

The healthy worker effect may impact findings in this study. Healthy workers are more likely to remain employed in occupations with exposure to vapours, dust, gases, and fumes, as workers with chronic respiratory diseases may avoid these jobs, transfer to less exposed jobs, or leave the workforce. This would result in an attenuation of the results; however elevated risks were still observed across many jobs. We also acknowledge that the issue of multiple comparisons may lead to some chance findings.

A key strength of this study is the use of a large, linked cohort of workers with enough power to examine risk of COPD in a wide range of occupations and by sex. This study reported on three distinct levels of occupation to identify risk of COPD which is likely not possible in smaller cohort designs. This speaks to the large size of the surveillance system with the ability to conduct detailed analyses. Results shown in this study are somewhat consistent with existing previous studies but also demonstrate new findings among female workers which is often limited in existing studies.

Overall, this study identified elevated risks of COPD for both male and female workers in various occupational groups and identified new associations among female workers, demonstrating the power of the study and the large cohort of workers. This study emphasizes the need to examine risk of COPD by sex with better understanding of factors contributing to risk differences. This study highlights the need for robust occupational surveillance of COPD in the working population.

### Supplementary Information

Supplementary Tables.

## Data Availability

The full dataset generated is available in Supplementary Tables 1 and 2. Additional supporting data may be available upon request if conditions comply with organization reporting guidelines and provincial and federal privacy regulations.
