# Supplementary material for: Risk of chronic obstructive pulmonary disease in a large cohort of Ontario, Canada workers
Source: Sci Rep. 2024 Apr 16;14:8756. doi: 10.1038/s41598-024-59429-1 (PMC11021393; doi:10.1038/s41598-024-59429-1)
Supplement: Supplementary file 1 — Supplementary Tables. [file 41598_2024_59429_MOESM1_ESM.docx]

**Supplementary Table 1. Risk of COPD by minor level occupation group in males and females in the ODSS (1983-2020)**

|  | **Males** |  | **Females** |  |
| --- | --- | --- | --- | --- |
| **[CCDO Minor Level] Occupation Groups** | **Cases** | **HR* (95% CI)** | **Cases** | **HR* (95% CI)** |
| **Managers and Administration** |  |  |  |  |
| [1113] Government administrators | 6 | 0.54 (0.23-1.31) | 8 | 0.74 (0.37-1.48) |
| [1116] Inspectors and regulatory officers, government | 18 | *0.52 (0.33-0.82)* | 9 | 0.59 (0.30-1.13) |
| [1119] Officials and administrators unique to government, n.e.c. | 16 | *0.59 (0.36-0.96)* | 24 | *0.53 (0.36-0.79)* |
| [1130] General managers and other senior officials | 6 | 0.65 (0.29-1.45) | <6 | -- |
| [1131] Management, natural sciences and engineering | <6 | -- | 6 | 1.25 (0.52-3.00) |
| [1133] Administrators in teaching and related fields | <6 | *--* | 10 | *0.43 (0.23-0.81)* |
| [1137] Sales and advertising management | <6 | -- | 11 | 1.34 (0.74-2.42) |
| [1142] Services management | 8 | 0.88 (0.44-1.77) | <6 | -- |
| [1143] Production management | 14 | *0.58 (0.34-0.97)* | 7 | 1.56 (0.74-3.27) |
| [1145] Management, construction operations | 18 | 1.12 (0.70-1.78) | <6 | -- |
| [1147] Management, transport and communications operations | 7 | 1.01 (0.48-2.12) | <6 | -- |
| [1149] Other Managers and Administrators, n.e.c. | 59 | 1.03 (0.80-1.33) | 42 | 0.88 (0.65-1.19) |
| [1171] Accountants, auditors and other financial officers | 15 | 0.70 (0.42-1.17) | 17 | *0.50 (0.31-0.81)* |
| [1174] Personnel and related officers | 6 | 0.86 (0.39-1.92) | 8 | *0.43 (0.21-0.86)* |
| [1176] Inspectors and regulatory officers, non-government | 22 | *0.59 (0.39-0.89)* | 17 | 0.97 (0.60-1.56) |
| [1179] Related to management and administration, n.e.c. | 32 | 0.93 (0.66-1.32) | 81 | *0.80 (0.64-0.99)* |
| **Natural Sciences, Engineering, Mathematics** |  |  |  |  |
| [2117] Physical sciences technologists and technicians | 36 | 0.76 (0.55-1.05) | 10 | 1.07 (0.57-1.98) |
| [2131] Agriculturists and related scientists | 16 | 0.82 (0.50-1.35) | <6 | -- |
| [2135] Life sciences technologists and technicians | 10 | 0.67 (0.36-1.25) | <6 | -- |
| [2139] Life sciences, n.e.c. | 8 | 1.01 (0.51-2.02) | <6 | -- |
| [2143] Civil engineers | 13 | 0.74 (0.43-1.28) | <6 | -- |
| [2144] Electrical engineers | 10 | 0.56 (0.30-1.04) | <6 | -- |
| [2145] Industrial engineers | 10 | 0.69 (0.37-1.29) | 7 | 1.22 (0.58-2.56) |
| [2147] Mechanical engineers | 13 | 0.71 (0.41-1.23) | <6 | -- |
| [2161] Surveyors | 29 | 0.79 (0.55-1.13) | <6 | -- |
| [2163] Draughtsmen | 9 | 0.62 (0.32-1.19) | <6 | -- |
| [2165] Architectural and engineering technologists and technicians | 161 | *0.82 (0.70-0.96)* | 39 | 1.02 (0.74-1.40) |
| [2169] Other in architecture and engineering, n.e.c. | 35 | 1.25 (0.90-1.75) | <6 | -- |
| [2183] Systems analysts, computer programmers and related | 21 | 0.70 (0.45-1.07) | 19 | 0.76 (0.49-1.20) |
| **Social Sciences and Related** |  |  |  |  |
| [2311] Economists | <6 | -- | 8 | 1.24 (0.62-2.47) |
| [2315] Psychologists | 12 | 1.49 (0.85-2.63) | 28 | 1.13 (0.78-1.63) |
| [2331] Social workers | 24 | 0.86 (0.57-1.28) | 83 | 0.90 (0.73-1.12) |
| [2333] Welfare and community services | 74 | 0.83 (0.66-1.05) | 281 | 1.03 (0.92-1.16) |
| [2339] Social work and related fields, n.e.c. | 6 | 1.24 (0.56-2.76) | 15 | 1.06 (0.64-1.75) |
| [2359] Library, museum and archival sciences, n.e.c. | <6 | -- | 7 | *0.41 (0.19-0.85)* |
| [2391] Educational and vocational counsellors | <6 | -- | 9 | 0.67 (0.35-1.29) |
| [2399] Social sciences and related fields, n.e.c. | 8 | 0.64 (0.32-1.29) | 39 | 0.92 (0.67-1.26) |
| **Teaching and Related** |  |  |  |  |
| [2719] University teaching and related, n.e.c. | <6 | -- | 17 | *0.45 (0.28-0.73)* |
| [2731] Elementary and kindergarten teachers | 39 | *0.35 (0.26-0.48)* | 137 | *0.28 (0.24-0.34)* |
| [2733] Secondary school teachers | 26 | *0.31 (0.21-0.46)* | 48 | *0.32 (0.24-0.42)* |
| [2739] Elementary and secondary school teaching and related, n.e.c. | 15 | *0.52 (0.31-0.86)* | 121 | *0.48 (0.40-0.57)* |
| [2791] Community college and vocational school-teachers | 15 | 1.13 (0.68-1.88) | 10 | 0.61 (0.33-1.13) |
| [2795] Teachers of exceptional students, n.e.c. | 6 | 1.49 (0.62-3.58) | 10 | 0.59 (0.32-1.10) |
| [2799] Other teaching and related, n.e.c. | 10 | 0.77 (0.41-1.43) | 10 | *0.42 (0.23-0.79)* |
| **Medicine and Health** |  |  |  |  |
| [3130] Supervisors: nursing | <6 | -- | 9 | *0.49 (0.26-0.95)* |
| [3131] Nurses, registered, graduate and nurses-in-training | 31 | *0.57 (0.40-0.82)* | 482 | *0.49 (0.45-0.54)* |
| [3134] Nursing assistants | 36 | 1.01 (0.73-1.40) | 382 | 0.96 (0.87-1.06) |
| [3135] Nursing aides and orderlies | 146 | 1.14 (0.97-1.34) | 1112 | **1.09 (1.03-1.16)** |
| [3137] Physiotherapists, occupational and other therapists | <6 | -- | 8 | *0.26 (0.13-0.51)* |
| [3139] Nursing, therapy and related assisting, n.e.c. | 72 | 0.56 (0.44-0.70) | 191 | 0.99 (0.85-1.14) |
| [3152] Dietitians and nutritionists | <6 | -- | 10 | 0.95 (0.51-1.76) |
| [3156] Medical laboratory technologists and technicians | 17 | 0.65 (0.41-1.05) | 87 | *0.69 (0.56-0.85)* |
| [3159] Other in medicine and health, n.e.c. | 10 | 0.71 (0.38-1.31) | 37 | 0.73 (0.53-1.00) |
| **Artistic, Literacy, and Recreational** |  |  |  |  |
| [3319] Fine and commercial art, photography and related fields, n.e.c. | 13 | 1.26 (0.73-2.16) | <6 | -- |
| [3339] Performing and audio-visual arts, n.e.c. | 31 | 1.20 (0.84-1.70) | <6 | -- |
| [3352] Writers and editors | 6 | 1.34 (0.60-2.99) | 6 | 1.13 (0.51-2.51) |
| [3370] Coaches, trainers, instructors and managers, sport and recreation | 7 | *0.42 (0.20-0.89)* | 10 | *0.50 (0.27-0.94)* |
| [3375] Attendants, sport and recreation | 59 | 0.97 (0.75-1.26) | 55 | **1.74 (1.34-2.27)** |
| **Clerical and Related** |  |  |  |  |
| [4111] Secretaries and stenographers | <6 | -- | 104 | *0.53 (0.43-0.64)* |
| [4113] Typists and clerk-typists | <6 | -- | 57 | 1.15 (0.89-1.50) |
| [4130] Supervisors: bookkeeping, account-recording and related | <6 | -- | 15 | 1.34 (0.81-2.22) |
| [4131] Bookkeepers and accounting clerks | 16 | 0.91 (0.55-1.48) | 70 | 0.80 (0.63-1.01) |
| [4133] Tellers and cashiers | 30 | 0.84 (0.59-1.20) | 549 | **1.37 (1.26-1.50)** |
| [4135] Insurance, bank and other finance clerks | <6 | -- | 9 | 0.76 (0.40-1.46) |
| [4137] Statistical clerks | 13 | 0.85 (0.49-1.47) | 9 | 0.93 (0.48-1.78) |
| [4139] Bookkeeping, account-recording and related, n.e.c. | 8 | 1.38 (0.69-2.77) | 36 | *0.70 (0.50-0.97)* |
| [4141] Office machine operators | 10 | 1.55 (0.83-2.88) | 10 | 1.26 (0.68-2.34) |
| [4143] Electronic data-processing equipment operators | 13 | 0.79 (0.46-1.36) | 61 | 0.81 (0.63-1.05) |
| [4150] Supervisors: material recording, scheduling and distributing | 58 | 1.04 (0.80-1.34) | 14 | 0.96 (0.57-1.62) |
| [4151] Production clerks | 109 | **1.34 (1.11-1.62)** | 53 | **1.45 (1.11-1.90)** |
| [4153] Shipping and receiving clerks | 884 | **1.14 (1.07-1.22)** | 190 | **1.54 (1.33-1.77)** |
| [4155] Stock clerks and related | 353 | 1.05 (0.94-1.16) | 121 | 1.13 (0.94-1.35) |
| [4157] Weighers | 17 | **2.24 (1.39-3.60)** | 6 | **2.84 (1.18-6.79)** |
| [4159] Material recording, scheduling and distributing, n.e.c. | 55 | 1.22 (0.94-1.59) | 44 | **1.80 (1.34-2.42)** |
| [4161] Library and file clerks | 8 | 1.64 (0.82-3.28) | 28 | 0.82 (0.57-1.19) |
| [4170] Supervisors: reception, information, mail and message distribution | 6 | 0.51 (0.23-1.14) | 11 | 1.41 (0.78-2.55) |
| [4171] Receptionists and information clerks | <6 | -- | 65 | 0.91 (0.71-1.16) |
| [4172] Mail carriers | 250 | *0.77 (0.68-0.87)* | 118 | 1.02 (0.85-1.22) |
| [4173] Mail and postal clerks | 157 | 0.90 (0.77-1.06) | 186 | **1.19 (1.03-1.37)** |
| [4175] Telephone operators | <6 | -- | 10 | 0.72 (0.39-1.34) |
| [4177] Messengers | 132 | 1.08 (0.91-1.28) | 32 | 1.17 (0.82-1.65) |
| [4179] Reception, information, mail and message distribution , n.e.c. | 9 | **2.14 (1.12-4.12)** | 9 | 1.04 (0.54-1.99) |
| [4190] Supervisors: other clerical and related, n.e.c. | <6 | -- | 11 | 0.70 (0.39-1.26) |
| [4191] Collectors | 6 | 0.92 (0.41-2.05) | 14 | 1.46 (0.87-2.47) |
| [4193] Travel clerks, ticket, station and freight agents | 16 | *0.46 (0.28-0.75)* | 26 | *0.63 (0.43-0.93)* |
| [4194] Hotel clerks | <6 | -- | 14 | 1.32 (0.78-2.23) |
| [4195] Personnel clerks | <6 | -- | 11 | 1.41 (0.78-2.55) |
| [4197] General office clerks | 40 | 1.08 (0.79-1.47) | 213 | 0.87 (0.76-1.00) |
| [4199] Other clerical and related, n.e.c. | 133 | 0.99 (0.84-1.18) | 273 | 1.10 (0.97-1.24) |
| **Sales** |  |  |  |  |
| [5130] Supervisors: sales, commodities | 223 | *0.78 (0.69-0.89)* | 225 | *0.87 (0.76-0.99)* |
| [5131] Technical salesmen and related advisers | 6 | 0.56 (0.25-1.24) | <6 | -- |
| [5133] Commercial travelers | 30 | 1.05 (0.73-1.50) | 6 | 0.74 (0.33-1.64) |
| [5135] Salesmen and salespersons, commodities, n.e.c. | 503 | 1.07 (0.98-1.16) | 821 | **1.17 (1.09-1.25)** |
| [5137] Sales clerks, commodities | 258 | *0.85 (0.75-0.96)* | 379 | 1.03 (0.93-1.14) |
| [5145] Service station attendants | 61 | **1.78 (1.38-2.29)** | 23 | **2.78 (1.84-4.18)** |
| [5149] Sales: commodities, n.e.c. | <6 | -- | 10 | **1.96 (1.05-3.64)** |
| [5179] Sales: services, n.e.c. | 6 | 0.88 (0.40-1.96) | <6 | -- |
| [5191] Buyers, wholesale and retail trade | 18 | 1.36 (0.85-2.15) | 40 | 1.05 (0.77-1.43) |
| [5193] Driver-salesmen | 86 | 1.12 (0.91-1.39) | <6 | -- |
| [5199] Other sales, n.e.c. | 15 | 1.35 (0.81-2.24) | 25 | 1.33 (0.90-1.96) |
| **Services** |  |  |  |  |
| [6111] Fire-fighting | 188 | *0.59 (0.51-0.68)* | <6 | -- |
| [6112] Policemen and detectives, government | 192 | *0.40 (0.35-0.46)* | 28 | *0.47 (0.33-0.69)* |
| [6113] Policemen and investigators, private | 8 | 0.65 (0.33-1.30) | 7 | 1.19 (0.56-2.49) |
| [6115] Guards and watchmen | 300 | 0.93 (0.83-1.04) | 145 | **1.36 (1.16-1.61)** |
| [6119] Protective service, n.e.c. | 9 | 0.63 (0.33-1.22) | 8 | 1.06 (0.53-2.11) |
| [6120] Supervisors: food and beverage preparation and related service | 55 | *0.76 (0.58-0.99)* | 130 | 1.08 (0.91-1.28) |
| [6121] Chefs and cooks | 359 | 1.00 (0.90-1.11) | 458 | **1.44 (1.31-1.58)** |
| [6123] Bartenders | 27 | 0.95 (0.65-1.39) | 52 | **1.38 (1.05-1.81)** |
| [6125] Waiters, hostesses and stewards, food and beverage | 74 | 0.86 (0.68-1.08) | 516 | **1.48 (1.35-1.61)** |
| [6129] Food and beverage preparation and related service , n.e.c. | 149 | 0.97 (0.83-1.14) | 634 | **1.34 (1.24-1.45)** |
| [6130] Supervisors: occupations in lodging and other accommodation | 14 | 1.15 (0.68-1.94) | 54 | **1.36 (1.04-1.77)** |
| [6131] Managers: hotel, motel and other accommodation | 6 | 1.50 (0.67-3.33) | 20 | **2.02 (1.30-3.14)** |
| [6133] Chambermaids and housemen | <6 | -- | 50 | 1.02 (0.77-1.34) |
| [6135] Sleeping-car and baggage porters, and bellmen | 14 | 1.40 (0.83-2.36) | <6 | -- |
| [6139] Occupations in lodging and other accommodation, n.e.c. | 13 | 0.87 (0.51-1.50) | 14 | 1.59 (0.94-2.68) |
| [6143] Barbers, hairdressers and related | <6 | -- | 7 | *0.32 (0.15-0.66)* |
| [6145] Hostesses and stewards, except food and beverage | 12 | 0.65 (0.37-1.15) | 18 | *0.29 (0.19-0.47)* |
| [6147] Babysitters | <6 | -- | 9 | *0.43 (0.22-0.83)* |
| [6149] Personal service occupations, n.e.c. | 69 | 1.10 (0.87-1.39) | 721 | **1.13 (1.05-1.22)** |
| [6162] Laundering and dry cleaning | 41 | **1.35 (1.00-1.84)** | 105 | 1.20 (0.99-1.45) |
| [6165] Pressing occupations | 10 | 1.33 (0.72-2.48) | 35 | **1.40 (1.00-1.95)** |
| [6169] Apparel and furnishings service, n.e.c. | 15 | 1.29 (0.78-2.15) | 6 | 1.50 (0.68-3.35) |
| [6190] Supervisors: other service | 93 | 1.11 (0.91-1.36) | 52 | **1.51 (1.15-1.99)** |
| [6191] Janitors, charworkers and cleaners | 1678 | **1.31 (1.24-1.37)** | 1112 | **1.40 (1.31-1.49)** |
| [6198] Occupations in labouring and other elemental work, services | 341 | **1.32 (1.19-1.47)** | 263 | **1.31 (1.16-1.48)** |
| [6199] Other service, n.e.c. | 64 | 1.13 (0.88-1.44) | 26 | **1.69 (1.15-2.48)** |
| **Farming, Horticulture, and Animal Husbandry** |  |  |  |  |
| [7112] Farmers | 30 | 1.13 (0.79-1.62) | 8 | 1.11 (0.56-2.22) |
| [7180] Foremen: other farming, horticultural and animal husbandry | 34 | 0.90 (0.64-1.26) | 9 | 1.15 (0.60-2.20) |
| [7182] Farm workers | 346 | **1.26 (1.13-1.40)** | 101 | **1.24 (1.02-1.51)** |
| [7195] Nursery and related workers | 383 | **1.25 (1.13-1.38)** | 67 | 1.14 (0.90-1.45) |
| [7199] Other farming, horticultural and animal husbandry, n.e.c. | 40 | 1.10 (0.80-1.49) | 24 | 1.30 (0.87-1.95) |
| **Fishing, Hunting, and Trapping** |  |  |  |  |
| [7313] Fishermen: net, trap and line | 10 | 1.26 (0.68-2.35) | <6 | -- |
| [7319] Fishing, hunting, trapping and related, n.e.c. | 6 | **2.53 (1.05-6.07)** | <6 | -- |
| **Forestry and Logging** |  |  |  |  |
| [7511] Forestry conservation | 13 | 1.16 (0.68-2.00) | <6 | -- |
| [7513] Timber cutting and related | 164 | **1.44 (1.23-1.68)** | <6 | -- |
| [7517] Log hoisting, sorting, moving and related | 42 | 0.96 (0.71-1.30) | <6 | -- |
| [7518] Occupations in labouring and other elemental work, forestry and logging | 42 | **1.47 (1.09-1.99)** | <6 | -- |
| [7519] Forestry and logging, n.e.c. | 15 | 1.28 (0.77-2.12) | <6 | -- |
| **Mining and Quarrying** |  |  |  |  |
| [7710] Foremen: mining and quarrying including oil and gas field | 19 | 1.15 (0.73-1.80) | <6 | -- |
| [7713] Other rock and soil-drilling | 85 | 1.14 (0.92-1.41) | <6 | -- |
| [7717] Mining and quarrying: cutting, handling and loading | 144 | **1.27 (1.08-1.50)** | <6 | -- |
| [7718] Labouring and other elemental work, mining and quarrying including oil and gas fields | 56 | 1.27 (0.98-1.66) | <6 | -- |
| [7719] Mining and quarrying including oil and gas field, n.e.c. | 94 | 1.15 (0.94-1.41) | <6 | -- |
| **Processing (Mineral, Metal, Chemical)** |  |  |  |  |
| [8111] Crushing and grinding, mineral ores | 9 | 1.53 (0.80-2.94) | <6 | -- |
| [8118] Occupations in labouring and other elemental work, mineral ore treating | 19 | **1.63 (1.04-2.55)** | <6 | -- |
| [8130] Foremen: metal processing and related | 22 | 0.83 (0.55-1.26) | <6 | -- |
| [8131] Metal smelting, converting and refining furnacemen | 76 | **1.35 (1.08-1.69)** | <6 | -- |
| [8133] Metal heat treating | 16 | 1.55 (0.95-2.54) | <6 | -- |
| [8135] Metal rolling | 35 | 1.36 (0.98-1.90) | <6 | -- |
| [8137] Moulding, coremaking and metal casting | 206 | **1.34 (1.17-1.53)** | 19 | **1.70 (1.08-2.66)** |
| [8141] Metal extruding and drawing | 16 | 1.27 (0.78-2.07) | <6 | -- |
| [8143] Plating, metal spraying and related | 40 | 1.22 (0.90-1.67) | 8 | 1.74 (0.87-3.47) |
| [8146] Inspecting, testing, grading and sampling, metal processing | 13 | 1.49 (0.86-2.57) | <6 | -- |
| [8148] Labouring and other elemental work, metal processing | 141 | **1.38 (1.17-1.63)** | 14 | **2.28 (1.35-3.84)** |
| [8149] Metal processing and related, n.e.c. | 318 | **1.31 (1.18-1.47)** | 27 | **1.49 (1.02-2.17)** |
| [8150] Foremen: clay, glass and stone processing, forming and related | 6 | 0.86 (0.39-1.91) | <6 | -- |
| [8151] Furnacemen and kilnmen: clay, glass and stone | 17 | 1.31 (0.81-2.11) | <6 | -- |
| [8153] Separating, grinding, crushing and mixing: clay, glass and stone | 13 | 0.94 (0.54-1.61) | <6 | -- |
| [8155] Forming: clay, glass and stone | 45 | 1.22 (0.91-1.63) | 8 | **2.77 (1.39-5.54)** |
| [8156] Inspecting, testing, grading and sampling: clay, glass and stone processing and forming | <6 | -- | 8 | 1.61 (0.80-3.22) |
| [8158] Labouring and other elemental work: clay, glass and stone processing and forming | 122 | **1.43 (1.19-1.70)** | 12 | 1.43 (0.81-2.51) |
| [8159] Clay, glass and stone processing, forming and related, n.e.c. | 71 | 1.19 (0.94-1.50) | 19 | **1.95 (1.24-3.05)** |
| [8160] Foremen: chemicals, petroleum, rubber, plastic and related materials processing | 26 | 1.01 (0.69-1.48) | <6 | -- |
| [8161] Mixing and blending, chemicals and related materials | 104 | **1.59 (1.31-1.93)** | 13 | **2.01 (1.16-3.46)** |
| [8171] Crushing and grinding, chemicals and related materials | 22 | 1.28 (0.84-1.94) | <6 | -- |
| [8173] Coating and calendering , chemicals and related materials | 10 | 0.92 (0.49-1.70) | 6 | 1.40 (0.63-3.11) |
| [8176] Inspecting, testing, grading and sampling: chemicals, petroleum, rubber, plastic and related materials processing | 7 | 1.12 (0.53-2.34) | 9 | 1.56 (0.81-3.00) |
| [8178] Labouring and other elemental work: chemicals, petroleum, rubber, plastic and related materials processing | 146 | **1.35 (1.15-1.59)** | 78 | **1.37 (1.10-1.71)** |
| [8179] Chemicals, petroleum, rubber, plastic and related materials processing, n.e.c. | 461 | 1.07 (0.98-1.17) | 308 | **1.37 (1.23-1.54)** |
| **Processing (Food, Wood, Textile)** |  |  |  |  |
| [8210] Foremen: food, beverage and related processing | 22 | 0.73 (0.48-1.11) | 12 | 1.00 (0.57-1.76) |
| [8211] Flour and grain milling | 22 | 1.41 (0.93-2.15) | <6 | -- |
| [8213] Baking, confectionery making and related | 141 | **1.19 (1.01-1.40)** | 117 | **1.24 (1.03-1.48)** |
| [8215] Slaughtering and meat cutting, canning, curing and packing | 221 | 0.96 (0.84-1.10) | 59 | 1.10 (0.85-1.42) |
| [8217] Fish canning, curing and packing | 28 | 1.19 (0.82-1.72) | 26 | 1.40 (0.95-2.05) |
| [8221] Fruit and vegetable canning, preserving and packing | 9 | 1.24 (0.65-2.38) | 7 | 1.73 (0.82-3.63) |
| [8223] Milk processing | 10 | 0.55 (0.29-1.01) | <6 | -- |
| [8226] Inspecting, testing, grading and sampling: food, beverage and related processing | 17 | 1.03 (0.64-1.65) | 14 | 0.85 (0.50-1.44) |
| [8227] Beverage processing | 39 | 0.94 (0.69-1.29) | 8 | 0.98 (0.49-1.96) |
| [8228] Labouring and other elemental work: food, beverage and related processing | 436 | **1.12 (1.02-1.23)** | 317 | **1.17 (1.04-1.30)** |
| [8229] Food, beverage and related processing, n.e.c. | 137 | 0.95 (0.81-1.13) | 92 | 1.22 (0.99-1.50) |
| [8231] Sawmill sawyers and related | 49 | **1.48 (1.12-1.96)** | <6 | -- |
| [8236] Inspecting, testing, grading and sampling: wood processing, except pulp and papermaking | 10 | 1.22 (0.66-2.27) | <6 | -- |
| [8238] Labouring and other elemental work: wood processing, except pulp and papermaking | 93 | **1.45 (1.18-1.78)** | 7 | 1.06 (0.51-2.23) |
| [8239] Wood processing, except pulp and papermaking, n.e.c. | 33 | **1.42 (1.01-1.99)** | 6 | 1.50 (0.67-3.34) |
| [8250] Foremen: pulp and papermaking and related | 8 | 1.11 (0.55-2.22) | <6 | -- |
| [8251] Cellulose pulp preparing | 10 | 0.90 (0.48-1.67) | <6 | -- |
| [8253] Papermaking and finishing | 50 | **1.39 (1.05-1.84)** | 11 | 1.22 (0.68-2.21) |
| [8258] Labouring and other elemental work, pulp and papermaking | 62 | **1.84 (1.43-2.36)** | <6 | -- |
| [8259] Pulp and papermaking and related, n.e.c. | 32 | 0.92 (0.65-1.29) | <6 | -- |
| [8263] Textile spinning and twisting | <6 | -- | 10 | 1.37 (0.74-2.56) |
| [8265] Textile winding and reeling | <6 | -- | 11 | **2.10 (1.16-3.79)** |
| [8267] Textile weaving | 18 | 0.95 (0.60-1.51) | 13 | 0.79 (0.46-1.36) |
| [8271] Knitting | 16 | 1.47 (0.90-2.40) | 21 | **1.63 (1.06-2.50)** |
| [8273] Textile bleaching and dyeing | 11 | 1.71 (0.95-3.09) | <6 | -- |
| [8275] Textile finishing and calendering | 14 | **2.09 (1.24-3.53)** | <6 | -- |
| [8276] Inspecting, testing, grading and sampling, textile processing | <6 | -- | 10 | 1.15 (0.62-2.14) |
| [8278] Labouring and other elemental work, textile processing | 59 | **1.46 (1.13-1.88)** | 32 | 1.04 (0.74-1.47) |
| [8279] Textile processing, n.e.c. | 46 | 1.41 (1.06-1.89) | 19 | 1.21 (0.77-1.90) |
| [8295] Hide and pelt processing | 14 | **2.93 (1.74-4.95)** | <6 | -- |
| [8298] Labouring and other elemental work, other processing | 157 | **1.48 (1.26-1.73)** | 40 | **1.46 (1.07-2.00)** |
| **Machining and Related** |  |  |  |  |
| [8310] Foremen: metal machining | 30 | 0.93 (0.65-1.34) | <6 | 2.37 (0.98-5.68) |
| [8311] Tool and die making | 171 | *0.77 (0.66-0.89)* | 8 | 1.00 (0.50-2.00) |
| [8313] Machinist and machine tool setting-up | 444 | *0.82 (0.75-0.90)* | 38 | 1.30 (0.95-1.79) |
| [8315] Machine tool operating | 206 | 1.06 (0.92-1.21) | 39 | **1.90 (1.38-2.59)** |
| [8319] Metal machining , n.e.c. | 7 | 0.77 (0.37-1.61) | <6 | -- |
| [8330] Foremen: metal shaping and forming, except machining | 38 | 0.81 (0.59-1.12) | <6 | -- |
| [8331] Forging | 62 | 1.08 (0.84-1.39) | 11 | **2.32 (1.29-4.20)** |
| [8333] Sheet metal workers | 279 | 1.01 (0.90-1.14) | 31 | **1.58 (1.11-2.25)** |
| [8334] Metalworking-machine operators, n.e.c. | 1429 | **1.17 (1.10-1.23)** | 416 | **1.52 (1.38-1.68)** |
| [8335] Welding and flame cutting | 1247 | **1.18 (1.11-1.25)** | 61 | **1.38 (1.07-1.77)** |
| [8337] Boilermakers, platers and structural metal workers | 116 | **1.25 (1.04-1.50)** | 11 | 1.40 (0.78-2.53) |
| [8339] Metal shaping and forming, except machining, n.e.c. | 282 | **1.31 (1.16-1.47)** | 58 | **1.54 (1.19-2.00)** |
| [8353] Wood sawing and related, except sawmill | 54 | **1.40 (1.07-1.83)** | <6 | -- |
| [8355] Planing, turning, shaping and related wood machining | 117 | 1.05 (0.88-1.26) | 16 | 0.86 (0.53-1.40) |
| [8359] Wood machining , n.e.c. | 10 | 1.33 (0.72-2.48) | <6 | -- |
| [8371] Cutting and shaping: clay, glass and stone | 15 | 1.07 (0.65-1.78) | <6 | -- |
| [8373] Abrading and polishing: clay, glass and stone, n.e.c. | 14 | **2.46 (1.46-4.16)** | <6 | -- |
| [8379] Clay, glass and stone and related materials machining, n.e.c. | 20 | 1.16 (0.75-1.80) | <6 | -- |
| [8391] Engravers, etchers and related | 14 | 0.76 (0.45-1.28) | <6 | -- |
| [8393] Filing, grinding, buffing, cleaning and polishing, n.e.c. | 267 | **1.32 (1.17-1.49)** | 31 | **1.75 (1.23-2.49)** |
| [8395] Patternmakers and mouldmakers, n.e.c. | 23 | *0.60 (0.40-0.90)* | 15 | **2.66 (1.60-4.42)** |
| [8396] Inspecting, testing, grading and sampling, machining, n.e.c. | 15 | 1.07 (0.64-1.77) | <6 | -- |
| [8399] Other machining and related, n.e.c. | 10 | 1.53 (0.82-2.84) | <6 | -- |
| **Product Fabricating, Assembling, and Repairing** |  |  |  |  |
| [8510] Foremen: Fabricating and assembling, metal products, n.e.c. | 52 | 0.77 (0.58-1.01) | 12 | 1.68 (0.96-2.97) |
| [8511] Engine and related equipment fabricating and assembling, n.e.c. | 28 | 1.45 (1.00-2.09) | <6 | -- |
| [8513] Motor vehicle fabricating and assembling, n.e.c. | 1175 | **1.07 (1.01-1.14)** | 378 | **1.40 (1.26-1.55)** |
| [8515] Aircraft fabricating and assembling, n.e.c. | 59 | *0.64 (0.49-0.82)* | 12 | 1.08 (0.61-1.90) |
| [8523] Industrial, farm, construction and other mechanized equipment and machinery fabricating and assembling, n.e.c. | 169 | **1.23 (1.05-1.43)** | 39 | **1.39 (1.01-1.90)** |
| [8526] Inspecting, testing, grading and sampling, fabricating and assembling metal products, n.e.c. | 79 | 1.04 (0.84-1.30) | 22 | **1.70 (1.12-2.58)** |
| [8528] Labouring and other elemental work, fabricating and assembling metal products, n.e.c. | 166 | **1.42 (1.22-1.66)** | 29 | **2.03 (1.41-2.92)** |
| [8529] Other fabricating and assembling, metal products, n.e.c. | 363 | **1.23 (1.11-1.36)** | 197 | **1.52 (1.32-1.75)** |
| [8530] Foremen: fabricating, assembling, installing and repairing, electrical, electronic and related equipment | 36 | 0.88 (0.63-1.22) | <6 | 0.86 (0.36-2.06) |
| [8531] Electrical equipment fabricating and assembling | 200 | 0.89 (0.78-1.03) | 170 | **1.30 (1.12-1.51)** |
| [8533] Electrical and related equipment installing and repairing, n.e.c. | 131 | *0.77 (0.65-0.92)* | 7 | 0.92 (0.44-1.93) |
| [8534] Electronic equipment fabricating and assembling | 38 | 1.22 (0.89-1.68) | 66 | 1.12 (0.88-1.43) |
| [8535] Electronic and related equipment installing and repairing, n.e.c. | 64 | *0.69 (0.54-0.88)* | 59 | 1.14 (0.88-1.47) |
| [8536] Inspecting, testing, grading and sampling: fabricating, assembling, installing and repairing electrical, electronic and related equipment | 7 | 0.80 (0.38-1.68) | <6 | -- |
| [8537] Radio and television service repairmen | 17 | 1.12 (0.69-1.80) | <6 | -- |
| [8538] Labouring and other elemental work: fabricating, assembling, installing and repairing electrical, electronic and related equipment | 22 | 0.83 (0.54-1.26) | 20 | 1.41 (0.91-2.19) |
| [8539] Fabricating, assembling, installing and repairing: electrical, electronic and related equipment, n.e.c. | 36 | 1.03 (0.75-1.44) | 19 | 1.43 (0.91-2.24) |
| [8540] Foremen: fabricating, assembling and repairing, wood products | 11 | 0.83 (0.46-1.51) | <6 | -- |
| [8541] Cabinet and wood furniture makers | 166 | 0.88 (0.75-1.02) | 27 | 1.18 (0.81-1.73) |
| [8548] Labouring and other elemental work: fabricating, assembling and repairing, wood products | 188 | **1.51 (1.31-1.74)** | 24 | **1.50 (1.01-2.25)** |
| [8549] Fabricating, assembling and repairing, wood products, n.e.c. | 199 | **1.25 (1.09-1.44)** | 38 | **1.58 (1.15-2.17)** |
| [8551] Patternmaking, marking and cutting: textile, fur and leather products | 33 | 0.97 (0.69-1.37) | 20 | 0.77 (0.49-1.19) |
| [8561] Shoemaking and repairing | 14 | **1.80 (1.07-3.05)** | 20 | **1.87 (1.21-2.90)** |
| [8562] Upholsterers | 54 | 0.88 (0.67-1.15) | 15 | 1.17 (0.71-1.95) |
| [8563] Sewing machine operators, textile and similar materials | 45 | 1.07 (0.80-1.43) | 206 | *0.79 (0.69-0.91)* |
| [8568] Labouring and other elemental work: fabricating, assembling and repairing, textile, fur and leather products | 19 | 0.98 (0.62-1.53) | 25 | 1.18 (0.80-1.75) |
| [8569] Fabricating, assembling and repairing: textile, fur and leather products, n.e.c. | 47 | **1.38 (1.04-1.84)** | 58 | 1.20 (0.93-1.55) |
| [8570] Foremen: fabricating, assembling and repairing, rubber, plastic and related products | 23 | 0.83 (0.55-1.24) | 9 | 1.58 (0.82-3.05) |
| [8571] Bonding and cementing, rubber, plastic and related products | 112 | **1.34 (1.12-1.62)** | 18 | 1.30 (0.82-2.07) |
| [8573] Moulding, rubber, plastic and related products | 28 | 1.23 (0.85-1.78) | 13 | 1.41 (0.82-2.43) |
| [8576] Inspecting, testing, grading and sampling: fabricating, assembling and repairing, rubber, plastic and related products | 9 | 1.29 (0.67-2.48) | 6 | 1.69 (0.76-3.75) |
| [8578] Labouring and other elemental work: fabricating, assembling and repairing, rubber, plastic and related products | 100 | **1.41 (1.16-1.72)** | 28 | 1.07 (0.74-1.55) |
| [8579] Fabricating, assembling and repairing: rubber, plastic and related products, n.e.c. | 97 | **1.23 (1.01-1.51)** | 67 | **1.87 (1.47-2.38)** |
| [8580] Foremen: mechanics and repairmen, except electrical | 37 | 1.17 (0.85-1.62) | <6 | -- |
| [8581] Motor vehicle mechanics and repairmen | 1256 | 0.97 (0.92-1.03) | 37 | **1.93 (1.40-2.67)** |
| [8582] Aircraft mechanics and repairmen | 40 | *0.54 (0.40-0.74)* | <6 | -- |
| [8583] Rail transport equipment mechanics and repairmen | 39 | 1.05 (0.77-1.43) | <6 | -- |
| [8584] Industrial, farm and construction machinery mechanics and repairmen | 876 | *0.88 (0.82-0.94)* | 24 | 1.44 (0.96-2.14) |
| [8585] Business and commercial machine mechanics and repairmen | 16 | 0.78 (0.48-1.27) | <6 | -- |
| [8588] Precision instrument mechanics and repairmen | 8 | 0.65 (0.32-1.30) | <6 | -- |
| [8589] Mechanics and repairmen, except electrical, n.e.c. | 400 | 0.92 (0.83-1.01) | 18 | **1.87 (1.18-2.97)** |
| [8590] Foremen: product fabricating, assembling and repairing, n.e.c. | 20 | 0.82 (0.53-1.27) | 6 | 1.15 (0.52-2.56) |
| [8591] Jewelry and silverware fabricating, assembling and repairing | 8 | 1.66 (0.83-3.31) | <6 | -- |
| [8592] Marine craft fabricating, assembling and repairing | 22 | 1.42 (0.93-2.15) | <6 | -- |
| [8593] Paper product fabricating and assembling | 144 | 1.15 (0.97-1.35) | 55 | **1.62 (1.24-2.11)** |
| [8595] Painting and decorating, except construction | 317 | **1.25 (1.12-1.40)** | 56 | **1.92 (1.48-2.50)** |
| [8598] Labouring and other elemental work: product fabricating, assembling and repairing, n.e.c. | 69 | **1.40 (1.10-1.77)** | 10 | 0.83 (0.44-1.53) |
| [8599] Other product fabricating, assembling and repairing, n.e.c. | 384 | 1.10 (1.00-1.22) | 260 | **1.35 (1.19-1.53)** |
| **Construction Trades** |  |  |  |  |
| [8710] Foremen: excavating, grading, paving and related | 38 | 0.97 (0.70-1.33) | <6 | -- |
| [8711] Excavating, grading and related | 247 | **1.34 (1.18-1.52)** | 9 | **2.23 (1.16-4.29)** |
| [8713] Paving, surfacing and related | 12 | 1.29 (0.73-2.26) | <6 | -- |
| [8715] Railway sectionmen and trackmen | 54 | 1.13 (0.87-1.48) | <6 | -- |
| [8718] Labouring and other elemental work: excavating, grading and paving | 119 | **1.75 (1.46-2.09)** | <6 | -- |
| [8719] Excavating, grading, paving and related, n.e.c. | 129 | **1.37 (1.15-1.63)** | <6 | -- |
| [8730] Foremen: electrical power, lighting and wire communications equipment erecting, installing and repairing | 31 | 0.85 (0.60-1.22) | <6 | -- |
| [8731] Electrical power linemen and related | 75 | *0.76 (0.61-0.96)* | <6 | -- |
| [8733] Construction electricians and repairmen | 313 | *0.63 (0.56-0.71)* | <6 | -- |
| [8735] Wire communications and related equipment installing and repairing | 97 | *0.56 (0.46-0.68)* | 9 | 0.68 (0.35-1.31) |
| [8738] Labouring and other elemental work: electrical power, lighting and wire communications equipment erecting, installing and repairing | 39 | 1.12 (0.82-1.53) | <6 | -- |
| [8739] Electrical power, lighting and wire communications equipment erecting, installing and repairing, n.e.c. | 49 | *0.62 (0.46-0.81)* | <6 | -- |
| [8780] Foremen: other construction trades | 140 | 0.98 (0.83-1.15) | <6 | -- |
| [8781] Carpenters and related | 584 | 0.99 (0.91-1.07) | 6 | 1.69 (0.76-3.77) |
| [8782] Brick and stone masons and tile setters | 189 | 1.12 (0.97-1.29) | <6 | -- |
| [8783] Concrete finishing and related | 69 | **1.49 (1.18-1.89)** | <6 | -- |
| [8784] Plasterers and related | 118 | 1.10 (0.92-1.32) | <6 | **--** |
| [8785] Painters, paperhangers and related | 225 | **1.56 (1.37-1.78)** | 14 | **2.05 (1.21-3.46)** |
| [8786] Insulating, construction | 42 | **1.72 (1.27-2.32)** | <6 | -- |
| [8787] Roofing, waterproofing and related | 184 | **1.64 (1.42-1.90)** | <6 | -- |
| [8791] Pipefitting, plumbing and related | 355 | 0.95 (0.85-1.05) | 9 | **2.38 (1.24-4.57)** |
| [8793] Structural metal erectors | 88 | **1.30 (1.06-1.61)** | <6 | -- |
| [8795] Glaziers | 77 | **1.31 (1.05-1.64)** | <6 | -- |
| [8796] Inspecting, testing, grading and sampling, construction, except electrical | 10 | *0.49 (0.27-0.92)* | <6 | -- |
| [8798] Labouring and other elemental work, other construction trades | 1086 | **1.46 (1.37-1.55)** | 11 | 1.32 (0.73-2.39) |
| [8799] Other construction trades, n.e.c. | 1101 | **1.41 (1.33-1.50)** | 28 | **1.90 (1.31-2.75)** |
| **Transport and Equipment Operating** |  |  |  |  |
| [9113] Air transport operating support | 40 | *0.55 (0.40-0.74)* | 8 | 0.70 (0.35-1.40) |
| [9119] Air transport operating, n.e.c. | 46 | *0.52 (0.39-0.70)* | 7 | *0.47 (0.22-0.98)* |
| [9130] Foremen: railway transport operating | 16 | 1.52 (0.93-2.48) | <6 | -- |
| [9131] Locomotive engineers and firemen | 25 | 0.98 (0.66-1.45) | <6 | -- |
| [9133] Conductors and brakemen, railway | 57 | 1.17 (0.90-1.52) | <6 | -- |
| [9135] Railway transport operating support | 28 | 1.09 (0.75-1.58) | <6 | -- |
| [9151] Deck officers | 11 | **1.95 (1.08-3.52)** | <6 | -- |
| [9153] Engineering officers, ship | 9 | 1.69 (0.88-3.25) | <6 | -- |
| [9155] Deck crew, ship | 37 | **1.74 (1.26-2.40)** | <6 | -- |
| [9157] Engine and boiler-room crew, ship | 9 | **2.34 (1.22-4.49)** | <6 | -- |
| [9159] Water transport operating, n.e.c. | 7 | 0.60 (0.28-1.25) | <6 | -- |
| [9170] Foremen: motor transport operating | 51 | 1.10 (0.84-1.45) | 8 | **3.04 (1.52-6.07)** |
| [9171] Bus drivers | 149 | 0.98 (0.84-1.16) | 92 | **1.49 (1.21-1.83)** |
| [9173] Taxi drivers and chauffeurs | 74 | 0.94 (0.75-1.18) | 24 | **1.67 (1.12-2.49)** |
| [9175] Truck drivers | 3793 | **1.43 (1.38-1.48)** | 208 | **1.87 (1.63-2.15)** |
| [9179] Motor transport operating, n.e.c. | 288 | **1.32 (1.17-1.48)** | 36 | **1.68 (1.21-2.34)** |
| [9191] Subway and street railway operating | 9 | 0.81 (0.42-1.56) | <6 | -- |
| [9193] Motormen and dinkeymen, except rail transport | 6 | 1.50 (0.67-3.34) | <6 | -- |
| [9199] Other transport and related equipment operating, n.e.c. | 311 | 1.07 (0.96-1.20) | 151 | **1.48 (1.26-1.74)** |
| **Materials Handling and Related** |  |  |  |  |
| [9310] Foremen: materials handling and related, n.e.c. | 66 | 1.20 (0.95-1.53) | 12 | 1.40 (0.79-2.46) |
| [9311] Hoisting, n.e.c. | 131 | 1.13 (0.95-1.34) | <6 | -- |
| [9313] Longshoremen, stevedores and freight handlers | 898 | **1.30 (1.21-1.38)** | 78 | **1.31 (1.04-1.63)** |
| [9315] Materials handling equipment operators, n.e.c. | 498 | **1.49 (1.37-1.63)** | 29 | **2.07 (1.44-2.98)** |
| [9317] Packaging occupations, n.e.c. | 323 | **1.27 (1.14-1.42)** | 397 | **1.31 (1.19-1.45)** |
| [9318] Labouring and other elemental work, materials handling | 1427 | **1.34 (1.27-1.42)** | 326 | **1.37 (1.23-1.53)** |
| [9319] Materials handling and related, n.e.c. | 333 | **1.25 (1.12-1.40)** | 48 | **1.36 (1.02-1.80)** |
| **Other Crafts and Equipment Operating** |  |  |  |  |
| [9510] Foremen: printing and related | 8 | 0.69 (0.35-1.38) | <6 | -- |
| [9511] Typesetters and compositors | 15 | 1.19 (0.72-1.98) | 6 | 0.91 (0.41-2.02) |
| [9512] Printing press | 172 | 1.01 (0.87-1.18) | 45 | **2.00 (1.49-2.67)** |
| [9514] Printing engravers, except photoengravers | 6 | 0.92 (0.41-2.05) | <6 | -- |
| [9515] Photoengravers and related | 15 | **1.78 (1.07-2.95)** | <6 | -- |
| [9517] Bookbinders and related | 56 | **1.40 (1.08-1.82)** | 65 | **1.79 (1.40-2.28)** |
| [9518] Labouring and other elemental work: printing and related, n.e.c. | 47 | **1.55 (1.17-2.07)** | 21 | **1.88 (1.23-2.89)** |
| [9519] Printing and related, n.e.c. | 83 | 1.03 (0.83-1.27) | 62 | **1.43 (1.11-1.83)** |
| [9531] Power station operators | 8 | *0.45 (0.22-0.90)* | <6 | -- |
| [9539] Stationary engine and utilities equipment operating and related, n.e.c. | 190 | **1.18 (1.02-1.36)** | 12 | 1.73 (0.98-3.05) |
| [9555] Sound recording and reproduction equipment operators | 7 | 1.79 (0.85-3.76) | <6 | -- |
| [9591] Photographic processing | 9 | 1.00 (0.52-1.92) | <6 | -- |
| [9910] Supervisors and foremen, n.e.c. | 83 | 0.97 (0.78-1.21) | 20 | **2.03 (1.31-3.15)** |
| [9916] Inspecting, testing, grading and sampling, n.e.c. | 47 | 1.02 (0.77-1.36) | 15 | 0.87 (0.52-1.44) |
| [9918] Labourers, n.e.c. | 3476 | **1.42 (1.37-1.47)** | 937 | **1.42 (1.33-1.52)** |
| [9919] Other occupations, n.e.c. | 967 | **1.16 (1.08-1.23)** | 171 | **1.34 (1.15-1.56)** |

-Statistically significant (α=0.05) increased risks are bolded, and statistically significant decreased risks are italicized

*-List of abbreviations* – COPD=chronic obstructive pulmonary disease, ODSS=occupational disease surveillance system, n.e.c.=not elsewhere classified
*Adjusted for birth year and age at start of follow up

x

**Supplementary Table 2. Risk of COPD by division and select major level occupation group in males and females in the ODSS (1983-2020) with and without the removal of workers who were previously diagnosed with asthma in the ODSS.**

|  | **Model 1 (asthma cases included)** | | | | **Model 2 (asthma cases removed)** | | | |
| --- | --- | --- | --- | --- | --- | --- | --- | --- |
|  | **Males** | | **Females** | | **Males** | | **Females** | |
| **Occupation Group (Division and Major levels)** | **Cases** | **HR (95% CI)^*^** | **Cases** | **HR (95% CI)^*^** | **Cases** | **HR (95% CI)^*^** | **Cases** | **HR (95% CI)^*^** |
| Managers and Administration | 245 | *0.72 (0.64-0.82)* | 258 | *0.68 (0.60-0.76)* | 158 | *0.67 (0.58-0.79)* | 131 | *0.64 (0.54-0.76)* |
| Natural Sciences, Engineering, Mathematics | 364 | *0.74 (0.67-0.82)* | 98 | 0.85 (0.69-1.03) | 247 | *0.73 (0.65-0.83)* | 55 | 0.90 (0.69-1.17) |
| Social Sciences and Related | 115 | *0.80 (0.67-0.96)* | 432 | 0.94 (0.86-1.04) | 67 | *0.68 (0.53-0.86)* | 214 | 0.88 (0.77-1.01) |
| Teaching and Related | 103 | 0.41 (0.34-0.50) | 330 | *0.35 (0.32-0.39)* | 67 | 0.39 (0.30-0.49) | 153 | *0.30 (0.26-0.36)* |
| Medicine and Health | 276 | *0.76 (0.68-0.86)* | 2028 | *0.77 (0.74-0.81)* | 166 | *0.67 (0.57-0.78)* | 1101 | *0.79 (0.74-0.84)* |
| Artistic, Literacy, and Recreational | 137 | 0.88 (0.75-1.05) | 90 | 0.91 (0.74-1.12) | 88 | 0.83 (0.67-1.02) | 49 | 0.95 (0.72-1.26) |
| Clerical and Related | 2125 | 1.02 (0.97-1.06) | 2162 | 1.04 (0.99-1.09) | 1411 | 0.98 (0.93-1.04) | 1099 | 0.99 (0.93-1.05) |
| Services | 3413 | 0.98 (0.95-1.02) | 3906 | **1.32 (1.27-1.37)** | 2199 | *0.92 (0.88-0.96)* | 2050 | **1.31 (1.25-1.38)** |
| Protective Services | 673 | *0.60 (0.56-0.65)* | 185 | 1.03 (0.89-1.19) | 444 | *0.58 (0.52-0.63)* | 95 | 1.02 (0.83-1.25) |
| Food And Beverage Preparation   and Related Services | 634 | 0.95 (0.88-1.03) | 1655 | **1.40 (1.33-1.47)** | 373 | 0.82 (0.74-0.91) | 859 | **1.38 (1.28-1.48)** |
| Sales | 1134 | 0.97 (0.91-1.03) | 1422 | **1.07 (1.01-1.13)** | 764 | 0.95 (0.89-1.03) | 763 | **1.09 (1.01-1.17)** |
| Farming, Horticulture, and Animal Husbandry | 799 | **1.23 (1.15-1.32)** | 197 | **1.19 (1.04-1.37)** | **534** | **1.20 (1.10-1.31)** | **122** | **1.42 (1.18-1.69)** |
| Farmers | 30 | 1.13 (0.79-1.62) | 8 | 1.11 (0.56-2.22) | 23 | 1.27 (0.84-1.91) | <6 | -- |
| Other Farming, Horticultural and   Animal Husbandry | 777 | **1.24 (1.15-1.33)** | 192 | **1.20 (1.04-1.39)** | 517 | **1.20 (1.10-1.31)** | 118 | **1.42 (1.18-1.70)** |
| Fishing, Hunting, and Trapping | 16 | 1.56 (0.95-2.54) | <6 | -- | 9 | 1.28 (0.67-2.46) | <6 | -- |
| Forestry and Logging | 247 | **1.29 (1.14-1.47)** | <6 | -- | 172 | **1.31 (1.13-1.53)** | <6 | -- |
| Mining and Quarrying | 330 | **1.23 (1.10-1.37)** | <6 | -- | 245 | **1.32 (1.17-1.50)** | <6 | -- |
| Processing (Mineral, Metal, Chemical) | 1735 | **1.24 (1.18-1.30)** | 501 | **1.39 (1.27-1.52)** | 1171 | **1.22 (1.15-1.29)** | 263 | **1.42 (1.18-1.70)** |
| Mineral Ore Treating | 34 | **1.54 (1.10-2.15)** | <6 | -- | 25 | **1.65 (1.11-2.44)** | <6 | -- |
| Metal Processing and Related | 786 | **1.30 (1.21-1.40)** | 65 | **1.40 (1.10-1.79)** | 556 | **1.35 (1.24-1.46)** | 35 | **1.43 (1.02-1.99)** |
| Clay Glass and Stone Processing   Forming and Related | 248 | **1.25 (1.10-1.42)** | 44 | **1.77 (1.32-2.38)** | 163 | **1.20 (1.03-1.40)** | 22 | **1.67 (1.10-2.54)** |
| Chemicals Petroleum Rubber   Plastic and Related Materials Processing | 712 | **1.13 (1.05-1.22)** | 397 | **1.35 (1.22-1.49)** | 456 | 1.06 (0.97-1.16) | 208 | **1.34 (1.17-1.54)** |
| Processing (Food, Wood, Textile) | 1556 | **1.15 (1.09-1.21)** | 774 | **1.17 (1.09-1.26)** | 1077 | **1.16 (1.10-1.24)** | 414 | **1.19 (1.08-1.31)** |
| Food and Beverage and Related   Processing | 931 | 1.03 (0.97-1.10) | 587 | **1.17 (1.07-1.27)** | 647 | 1.05 (0.97-1.13) | 316 | **1.19 (1.06-1.33)** |
| Wood Processing  Except Paper Pulp | 173 | **1.39 (1.20-1.62)** | 19 | 1.16 (0.74-1.81) | 120 | **1.42 (1.18-1.69)** | 9 | 1.04 (0.54-1.99) |
| Pulp and Papermaking and   Related | 154 | **1.31 (1.12-1.54)** | 17 | 0.98 (0.61-1.57) | 115 | **1.43 (1.19-1.72)** | 8 | 0.87 (0.43-1.73) |
| Textile Processing | 162 | **1.33 (1.14-1.55)** | 109 | 1.14 (0.95-1.38) | 106 | **1.27 (1.05-1.53)** | 60 | 1.19 (0.92-1.53) |
| Other Processing | 179 | **1.53 (1.32-1.78)** | 48 | **1.37 (1.03-1.82)** | 122 | **1.53 (1.28-1.83)** | 25 | 1.36 (0.92-2.01) |
| Machining and Related | 4191 | **1.09 (1.05-1.12)** | 708 | **1.49 (1.39-1.61)** | 2849 | **1.07 (1.03-1.12)** | 394 | **1.58 (1.42-1.74)** |
| Metal Machining | 797 | *0.84 (0.78-0.90)* | 96 | **1.59 (1.30-1.94)** | 560 | *0.86 (0.79-0.94)* | 55 | **1.72 (1.32-2.24)** |
| Metal Shaping and Forming   Except Machining | 3154 | **1.16 (1.12-1.20)** | 559 | **1.50 (1.38-1.64)** | 2135 | **1.14 (1.09-1.20)** | 305 | **1.55 (1.38-1.74)** |
| Wood Machining | 179 | 1.09 (0.94-1.26) | 28 | 1.09 (0.76-1.59) | 132 | 1.17 (0.99-1.39) | 16 | 1.18 (0.72-1.93) |
| Clay, Glass, Stone and Related   Materials Machining | 47 | 1.27 (0.96-1.69) | <6 | -- | 31 | 1.22 (0.86-1.74) | <6 | -- |
| Other Machining and Related | 325 | **1.17 (1.05-1.30)** | 56 | **1.68 (1.29-2.19)** | 210 | 1.10 (0.96-1.26) | 36 | **2.04 (1.47-2.83)** |
| Product Fabricating, Assembling, and Repairing | 6058 | 0.99 (0.96-1.02) | 1811 | **1.28 (1.21-1.34)** | 4192 | 1.00 (0.97-1.03) | 986 | **1.31 (1.23-1.41)** |
| Fabricating and Assembling Metal   Products, n.e.c. | 1852 | **1.08 (1.03-1.13)** | 637 | **1.44 (1.33-1.56)** | 1267 | **1.07 (1.01-1.14)** | 351 | **1.50 (1.35-1.67)** |
| Fabricating Assembling Installing   and Repairing Electrical and   Electronic and Related Equipment | 513 | *0.84 (0.77-0.92)* | 334 | **1.23 (1.11-1.38)** | 344 | *0.82 (0.74-0.92)* | 176 | **1.22 (1.05-1.42)** |
| Fabricating Assembling and   Repairing, Wood | 511 | **1.14 (1.05-1.25)** | 80 | **1.33 (1.07-1.66)** | 342 | **1.12 (1.01-1.25)** | 47 | **1.48 (1.11-1.97)** |
| Fabricating Assembling and   Repairing, Textile Fur and Leather | 197 | 1.01 (0.88-1.16) | 332 | *0.88 (0.79-0.98)* | 133 | 1.00 (0.84-1.18) | 181 | 0.90 (0.78-1.04) |
| Fabricating Assembling and   Repairing, Rubber, Plastic, and   Related | 342 | **1.28 (1.15-1.42)** | 136 | **1.49 (1.26-1.76)** | 232 | **1.27 (1.11-1.44)** | 71 | **1.46 (1.16-1.85)** |
| Mechanics and Repairers Except   Electrical | 2354 | *0.90 (0.86-0.94)* | 82 | **1.63 (1.31-2.03)** | 1658 | *0.93 (0.88-0.98)* | 45 | **1.70 (1.27-2.27)** |
| Other Product Fabricating   Assembling and Repairing | 934 | **1.16 (1.09-1.24)** | 387 | **1.42 (1.28-1.57)** | 631 | **1.14 (1.06-1.24)** | 211 | **1.46 (1.27-1.67)** |
| Construction Trades | 4820 | **1.15 (1.12-1.19)** | 126 | **1.54 (1.29-1.83)** | 3328 | **1.16 (1.12-1.21)** | 67 | **1.56 (1.22-1.98)** |
| Excavating Grading Paving and   Related | 555 | **1.35 (1.25-1.47)** | 16 | **1.67 (1.02-2.73)** | 383 | **1.36 (1.23-1.50)** | 7 | 1.40 (0.67-2.94) |
| Electrical Power Lighting and Wire   Communications Equipment   Erecting Installing and Repairing | 557 | *0.65 (0.60-0.71)* | 23 | 0.86 (0.57-1.29) | 367 | *0.62 (0.56-0.69)* | 10 | 0.70 (0.38-1.31) |
| Other Construction Trades | 3829 | **1.27 (1.23-1.31)** | 88 | **1.89 (1.54-2.34)** | 2660 | **1.29 (1.24-1.35)** | 50 | **2.06 (1.56-2.72)** |
| Transport and Equipment Operating | 4534 | **1.32 (1.28-1.37)** | 470 | **1.53 (1.40-1.68)** | 3135 | **1.34 (1.29-1.39)** | 214 | **1.31 (1.15-1.50)** |
| Air Transport Operating | 75 | *0.54 (0.43-0.67)* | 16 | *0.61 (0.37-0.99)* | 55 | *0.57 (0.44-0.75)* | 8 | 0.57 (0.29-1.15) |
| Railway Transport Operating | 116 | 1.17 (0.97-1.40) | 6 | 1.31 (0.59-2.91) | 87 | **1.27 (1.03-1.57)** | <6 | -- |
| Water Transport Operating | 68 | **1.49 (1.18-1.90)** | <6 | -- | 50 | **1.60 (1.21-2.11)** | <6 | -- |
| Motor Transport Operating | 4133 | **1.38 (1.34-1.43)** | 335 | **1.70 (1.53-1.90)** | 2856 | **1.39 (1.34-1.45)** | 153 | **1.46 (1.25-1.72)** |
| Other Transport and Related   Equipment Operating | 322 | 1.06 (0.95-1.19) | 153 | **1.49 (1.27-1.75)** | 201 | 0.96 (0.84-1.11) | 67 | 1.23 (0.97-1.57) |
| Materials Handling and Related | 3220 | **1.33 (1.28-1.38)** | 846 | **1.36 (1.26-1.45)** | 2165 | **1.30 (1.25-1.36)** | 454 | **1.37 (1.25-1.51)** |
| Other Crafts and Equipment Operating | 572 | **1.09 (1.01-1.19)** | 205 | **1.48 (1.29-1.70)** | 377 | 1.05 (0.95-1.16) | 108 | **1.47 (1.22-1.78)** |
| Printing and Related | 350 | 1.09 (0.98-1.21) | 190 | **1.59 (1.37-1.83)** | 227 | 1.03 (0.90-1.17) | 101 | **1.59 (1.30-1.93)** |
| Stationary Engine and Utilities   Equipment Operating and Related | 201 | 1.10 (0.96-1.26) | 13 | 1.46 (0.85-2.52) | 135 | 1.07 (0.90-1.27) | 6 | 1.27 (0.57-2.84) |

-Division level occupations are shown with a grey background, major level occupations are indented

-Statistically significant (α=0.05) increased risks are bolded, and statistically significant decreased risks are italicized

*-List of abbreviations* – COPD=chronic obstructive pulmonary disease, ODSS=occupational disease surveillance system, n.e.c.=not elsewhere classified
^*^Adjusted for birth year and age at start of follow up
